# Heart rate recovery in asthmastic children and adolescents after clinical field test

**DOI:** 10.1186/s12890-020-01355-9

**Published:** 2021-02-19

**Authors:** Élida Pereira Silva, Bruno Alvarenga Soares, Mariana M. Reimberg, Raphael Ritti-Dias, Karina Silva Nascimento, Fabiana Silvia Anjos, Gustavo Falbo Wandalsen, Dirceu Solé, Simone Dal Corso, Fernanda Cordoba Lanza

**Affiliations:** 1 Post Graduate Programa in Rehabilitation Sciences, Universidade Nove de Julho – UNINOVE, São Paulo, SP 01525-000 Brazil; 2 Graduate Program in Rehabilitation Sciences, Department of Physical Therapy, Universidade Federal de Minas Gerais (UFMG), Belo Horizonte, MG 31270-901 Brazil; 3 Pediatric Department, Universidade Federal de São Paulo – UNIFESP, São Paulo, SP 04025-002 Brazil

**Keywords:** Asthma, Modified shuttle test, Heart rate recovery

## Abstract

**Background:**

Inflammation caused by chronic lung disease in childhood may lead to delayed heart rate recovery (HRR) however, there is lack of evidence on HRR in this population. The aim was to assess HRR after functional capacity testing in asthmatic children and adolescents and to compare with severity and disease control.

**Method:**

This was a study secondary to a randomized control trial. The modified shuttle test (MST) was performed to assess functional capacity and HRR. This is an externally cadenced test in which the distance walked is the outcome. HRR was assessed after MST and was defined as HR at exercise peak minus HR in the second minute after the end of exercise. Asthma control was assessed by the Asthma Control Test (ACT). Data normality was tested by Shapiro Wilk and the comparison between groups was made by Student’s t test or Mann Whitney test for numerical variables, and by Chi-square test for categorical variables. Statistical significance was considered when *p* < 0.05. SPSS version 20 was used in the analyzes.

**Results:**

The sample included 77 patients diagnosed with asthma (asthma group - AG) who were regularly treated for asthma. Control group (CG) consisted of 44 volunteers considered healthy, matched in age and gender to AG. The median age of CG was 12 (10–14) years and in AG 11 (9–13 years) being classified as mild to moderate asthmatic, and 57% of the sample had controlled asthma by ACT. Distance walked in the CG was 952 ± 286 m and AG 799 ± 313 m, *p* = 0.001. HRR was more efficient in CG (79 ± 15 bpm) compared to AG (69 ± 12 bpm), p = 0.001. The mild (69 ± 12 beats) and severe (72 ± 15 beats) AG presented worse HRR compared to control group (79 ± 15 bpm), *p* < 0.05.

**Conclusions:**

Asthmatic children and adolescents have delayed HRR after modified Shuttle test compared to their peers, suggesting that asthma leads to autonomic nervous system imbalance.

**Trial registration:** Registered in Clinical Trials under number NCT02383069 and approved by the Universidade Nove de Julho - UNINOVE Research Ethics Committee, protocol number 738192/2014.

## Background

Heart rate recovery (HRR) is defined as the reduction in heart rate to baseline after a period of stress [1]. It reflects the balance of the autonomic nervous system, since the reduction in heart rate after exercise is related to the interaction of parasympathetic and sympathetic activity [1, 2]. Initially, the sudden fall in heart rate occurs through parasympathetic reactivation and the sympathetic system withdrawal reduces even further these values [1, 2].

Lower values of HRR after maximum [2] and submaximum exercise [3] reflect an autonomic nervous system imbalance [2]. This situation is associated with increased risk of cardiovascular and cardiometabolic events [4], and also mortality in chronic diseases [2].

In healthy children and adolescents, HRR was assessed after cardiopulmonary exercise testing (CPET) [5] and after clinical field tests, such as the 6-min walk test [6] and the 3-min step test [7]. The association of HRR with cardiometabolic risk has already been described in children [7] as well as in adults [4].

Sympathovagal imbalance was observed in individuals with asthma, however this dysfunction was assessed by the analysis of heart rate variability (HRV) [8, 9]. Both HRR and HRV methods are related to the autonomic nervous system [10]. However, HRV may be influenced by environmental factors (noise, light, temperature, etc.), while HRR is a simpler method, and is calculated in absolute heart rate values and no specific software is required for its use [1, 2]. To the best of our knowledge, no study has evaluated autonomic dysfunction through HRR in asthmatic children.

Asthma patients are known to have reduced functional capacity assessed by simple tests such as the Shuttle test [11]. Modified Shuttle test (MST) is an externally cadenced field test with maximum test characteristics [11, 12]. Because it is easy to perform and provides useful information on exercise capacity [12], MST is a promising test to evaluate HRR in asthmatic children.

Since asthma is a chronic inflammatory disease associated with the patient’s sedentary lifestyle [11], it is hypothesized that there is sympathovagal dysfunction and that it can be detected by HRR after a clinical field test. Thus, this study aims to evaluate HRR after functional capacity testing in asthmatic children and adolescents to compare with severity and disease control.

## Method

This was a study secondary to the a randomized clinical trial registered in Clinical Trials under number ClinicalTrials.gov Identifier: NCT02383069 and approved by the Universidade Nove de Julho - UNINOVE Research Ethics Committee, protocol number 738192/2014. The study was performed at the Rehabilitation Laboratory at Universidade Nove de Julho. Patients were from the Asthma Clinic of the Discipline of Allergy, Clinical Immunology and Rheumatology of the Department of Pediatrics of UNIFESP. After signing the Informed Consent Form by the parents / guardians and signing the Child / Adolescent Consent Form, the assessments were initiated.

The study consisted of two groups: asthma group (AG) and control group (CG). Children and adolescents from 6 to 17 years of age, of both sexes, under regular asthma treatment for at least 3 months and with adherence to medical treatment, based on physician appointment, with specialist follow-up, all the severities of GINA Steps [13] with no exacerbation in the last 4 weeks were included in the AG. In the CG, healthy volunteers without acute illnesses in the last 4 weeks were included, normal lung function (> 80% of normal value) [14] matched by age and sex to AG in a 2:1 ratio. Those born prematurely and unable to perform the proposed tests were excluded.

To evaluate the asthma severity, AG were split according to GINA as mild asthma (GINA Steps 1 and 2), moderate asthma (GINA Step 3) and severe asthma (GINA Steps 4 and 5), and compared to the control group. To evaluate the asthma control, the volunteers were split into controlled asthma (ACT / C-ACT > 19), and poor or uncontrolled asthma (ACT / C-ACT ≤19), and compared to the control group.

### Protocol

The evaluations took place between April 2014 and November 2015 in a single visit. The following procedures were performed: application of the asthma control questionnaire (ACT or C-ACT, spirometry (pre and post bronchodilator) and the modified Shuttle test (MST).

### Ashma assessment

The ACT - Asthma Control Test [15] and C-ACT - Child Asthma Control Test [16] questionnaires are questionnaires for assessing asthma control based on patient perception. It refers to activity limitation, dyspnea and nocturnal symptoms in the last 4 weeks. Answers are scored from one (worst) to five (best), with the highest score indicating better asthma control. The ACT questionnaire consists of five questions and the C-ACT, applied to children aged 4–11 years, consists of seven questions, four directed to children and three to caregivers. In both questionnaires, asthma was considered controlled when the score was greater than 19, and uncontrolled when the score was ≤19 [16].

### Lung function

Spirometry was performed using the equipment Ultima CPX (MedGraphics Corporation®, St. Paul, MN, USA). Acceptability and reproducibility criteria were in accordance with ATS / ERS recommendations [17]. The variables analyzed were forced vital capacity (FVC), forced expiratory volume in 1 s (FEV_1_), FEV_1_ / FVC ratio and forced expiratory flow between 25 and 75% of FVC (FEF_25–75_). AG volunteers performed a pre and post bronchodilator test (Salbutamol 400 μg – inhaled drug).

Anthropometric variables (weight and height) were evaluated to characterize the sample and to determine the lung function normality. Additionally, the body mass index (BMI) was used and eutrophic cut off based on Z-score [18].

### Modified shuttle test (MST)

MST was performed according to ERS / ATS recommendations [19]. This is an externally cadenced test in which the test speed increases each minute, from 1.79 to 10.2Km/h. It is performed in a 10 m corridor and volunteers are allowed to walk/run. The test was performed twice with an interval of 30 min between them. HR and SpO_2_ were measured at rest (before the start of the test), continuously during the MST, and after 2 min of the end of the test. The distance walked from the best test was used as an outcome and was represented in absolute values and as a percentage of predicted [20]. In AG, MST was performed at least 30 min after Salbutamol administration.

Heart rate was measured by the POLAR® Ft2 heart rate monitor positioned at the height of the sternum xiphoid process. The HRR was calculated as the HR peak (at the end of MST) minus the HR recovery (at the second minute after MST), (HRR = HR peak – HR recovery) as described in previous studies [1–5, 21, 22].

### Statistical analysis

Data normality was tested by Shapiro Wilk and presented as mean (standard deviation) or median (interquartile range 25–75) according to adherence to the Gauss curve.

The comparison between groups was made by Student’s t test or Mann Whitney test for numerical variables, and by Chi-square test for categorical variables.

To evaluate the asthma severity, AG were split according to GINA as mild asthma (GINA Steps 1 and 2 – *n* = 25), moderate asthma (GINA Step 3 – *n* = 30) and severe asthma (GINA Steps 4 and 5 – *n* = 22), and compared to the control group (*n* = 44). To evaluate the asthma control, the volunteers were split into controlled asthma (ACT / C-ACT > 19), and poor or uncontrolled asthma (ACT / C-ACT ≤19), and compared to the control group. These comparisons were made by one-way ANOVA or Kruskal Wallis according to normality.

Statistical significance was considered when *p* < 0.05. SPSS version 20 was used in the analyzes.

## Results

Eighty-four AG volunteers were recruited, seven of whom were excluded because they had no interest in participating or for other associated diseases, of which 77 were included. In the CG 48 were eligible, 4 excluded because they could not perform the tests and because of spirometry abnormalities; thus, 44 volunteers participated in the CG. The median age in AG was 11 (9–13 years), 78% of volunteers were eutrophic and 64% male, with normal lung function. Regarding the severity of the disease, *n* = 25 (32%) were GINA Steps 1 and 2; *n* = 30 (39%) were GINA Step 3 ; and *n* = 22 (29%) were GINA Steps 4 and 5. According to asthma control, 57% (44) of the sample had controlled asthma. Anthropometric characteristics and lung function are described in Table 1.

**Table 1:** Anthropometric and lung function for asthma and control groups

	AG (***n*** = 77)	CG (***n*** = 44)	***p***
Age, years	11 [9–13]	12 [10–14]	0.14
Children, n (%)	40 (52)	19 (43)	0.35
Gender (male), n (%)	49 (64)	24 (55)	0.32
BMI, Kg/m^**2**^	19 [16–23]	19 [17–24]	0.76
Eutrofic Z-score, n (%)	60 (78)	37 (84)	0.51
FVC, L (%pred)	2.6 [2.1–3.1] (103 [94–109])	2.8 [2.2–3.4] (109 [97–122])	0.23
FEV_1_, L (%pred)	2.3 [1.8–2.9] (96 [85–106])	2.4 [1.8–3.0] (102 [88–114])	0.28
FEV_1_/FVC	88 [82–92]	87 [80–92]	0.40
FEF _25–75%,_ L (%pred)	2.3 [1.8–3.2] (95 [71–116])	2.9 [2.1–3.8] (96 [82–127])	0.03

*BMI* body mass index, *kg/m*^*2*^ kilogram per square meter, *FVC* forced vital capacity, *%pred* percentage predicted for age and gender, *VEF*_*1*_ forced expiratory volume in the first second, *FEV*_*1*_*/FVC* ratio of forced expiratory volume in the first second divided by forced vital capacity, *FEF*_*25-75%*_ forced expiratory flow 25 to 75%

About functional capacity, the AG had shorter distance walked in the MST compared to the CG, *p* = 0.001 (Table 2). At rest, HR was statically higher in AG (93 ± 13 bpm) compared to CG (86 ± 13 bpm), *p* = 0.003, although at exercise peak there was no statistically significant different between groups, *p* = 0.06 (Table 2). HRR was worse in AG, with lower HR decrease after exercise (79 ± 15 bpm) compared to CG (69 ± 12 bpm), *p* = 0.001.

**Table 2:** Heart rate behavior and distance walked in the Shuttle Modified Test between groups

	AG (***n*** = 77)	CG (***n*** = 44)	***p***
Distance walked, meters (%pred)	799 ± 213 (83 ± 18)	952 ± 286 (95 ± 19)	0.001
Heart rate at rest, bpm	93 ± 13	86 ± 13	0.003
Heart rate at peak, bpm	187 [179–196]	192 [184–200]	0.06
Heart rate 2°min recovery, bpm	116 ± 16	112 ± 18	0.13
ΔHRR, beats	69 ± 12	79 ± 15	0.001

*ΔHRR* heart rate recovery delta (Heart rate at peak – heart rate at 2°minute recovery)

**Fig. 1:**
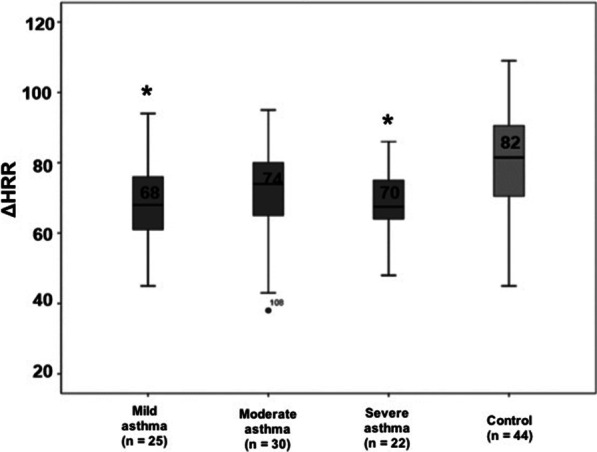
Heart rate recovery after Modified Shuttle test according to asthma severity. Mild asthma: GINA Steps 1 and 2; moderate asthma: GINA Step 3; and severe asthma: GINA Steps 4 and 5. * *p* = 0.01 vs control group

The AG was split into subgroups according to severity and compared to CG. Only the severe and mild asthma groups had lower ΔHRR compared to the CG (severe x control, *p* = 0.014 and mild x control, *p* = 0.013). (Fig. 1).

**Fig. 2:**
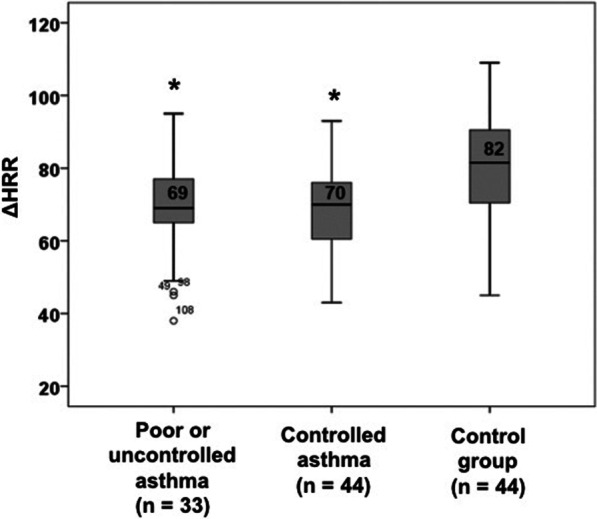
Heart rate recovery (ΔHRR) after Modified Shuttle test split according to asthma control by ACT / C-ACT score. Controlled asthma: ACT / C-ACT over 19, and poor or uncontrolled asthma: ACT / C-ACT ≤19. * *p* = 0.01 vs control group

Asthma control assessed by ACT and C-ACT influenced the ΔHRR. The control group showed better heart rate recovery when compared to controlled asthma group (> 19) and with partially or uncontrolled asthma group (≤19), *p* = 0.02. However, there was no difference between under control asthma group and partially or uncontrolled asthma group (*p* = 0.56) (Fig. 2).

In AG, the ΔHRR did not correlate significantly with distance walked (*r* = 0.09, *p* = 0.39), asthma severity (*r* = − 0.07, *p* = 0.53) and asthma control (*r* = 0.17, *p* = 0.11). Similarly, in the CG, distance walked had no statistically significant correlation with ΔHRR (*r* = 0.12, *p* = 0.40).

Considering a one-tailed curve, because the hypothesis was that asthma group had worse HRR, with p 0.05, *N* = 77 for asthma group and *N* = 44 for control group, the post hoc power was 83%. According to the asthma severity: mild (*n* = 25), moderate (*n* = 30), severe (*n* = 22) compared to the control group (*n* = 44), the post hoc power was 99%, with *p* = 0.05. According to the asthma control, controlled asthma (*n* = 33) poor or uncontrolled asthma (*n* = 44) compared to the control group (*n* = 44), the post hoc power was 98%, with *p* = 0 .05.

## Discussion

After assessed asthmatic children and adolescents, it was observed that HRR after modified shuttle test is worse when compared to healthy peers. Additionally, the severity of the disease impairs the recovery of heart rate. The HR recovery delay after exercise may be indicative of autonomic dysfunction caused by sympathovagal imbalance [2–4]. This is the first study to assess HRR autonomic dysfunction in asthmatic children and adolescents.

The severity of asthma (severe and mild) was related to worse HRR compared to the CG. Although there was no statistically difference of HRR in the moderate asthma group compared to CG, it is known that this difference is clinically important. Qiu et al. [4] describe in their meta-analysis that every 10 beats per minute reduced in heart rate recovery increases the risk of cardiovascular events by 13% and the risk of mortality by 9% compared to general population. Emin et al. [23] also assessed autonomic dysfunction in 77 asthmatic children, divided by the severity of the disease into mild, moderate and severe, through HRV. There was positive correlation between asthma severity and autonomic modulation, in other words, the higher parasympathetic dysfunction is related to disease severity. In our study, this parasympathetic dysfunction was demonstrated by the recovery of the most slowed HR in AG (severe and mild) compared to the control group.

Some studies reported the correlation between HRV and severity of the disease. Milagro et al. [8] analyzed HRV in preschoolers divided into 3 groups based on the risk of developing asthma (high and low risk) and the group treated with inhaled corticosteroids (confirmed asthma). This study also observed reduction in sympathovagal balance in the high-risk group, with results similar to the confirmed asthma group. This shows an intrinsic change in autonomic nervous system in disease groups. Gomes et al. [9] suggest a predominance of sympathetic activity during crisis, which could be explained by the greater release of inflammatory mediators during this period. In controlled asthmatics, they observed higher parasympathetic activity. In the present study, the recovery of heart rate suggests an increased sympathetic activity, even in volunteers out of the crisis period and in undergoing treatment.

Additionally, a variable described as a predictor of HRR is resting heart rate [5]. AG volunteers had higher resting HR compared to CG and slower HRR with statistically significant difference. This finding corroborates other previous studies [5, 7, 24, 25] that also observed slower HRR in volunteers with higher resting HR. Some hypotheses may explain this fact, such as the sedentary lifestyle [11] imposed by the disease, use of chronic medications and inhalation of salbutamol (400 μg) before the test, and also the inflammatory profile in chronic disease. Previous study in asthmatic children showed that medication increases heart rate by 13% and this effect lasts for 45 min [26].

According to disease control, our study observed no difference in HRR when compared to controlled and partially controlled asthmatics, different from Lufti’s study [27] in which controlled asthmatic adults demonstrated higher parasympathetic activation (higher HF component and lower LF / HF ratio) compared to uncontrolled asthma group, however, in a different population from our study.

Clinical field tests may be an easy alternative to CPET. There is positive association between HRR at step test and oxygen consumption evaluated in CPET [28]. Our study presents the possibility of evaluating HRR during MST, an easy to perform low cost test with a maximum assessment  [12, 20].

Although no study in children has defined abnormal values for HRR as already has been described for adults [2, 3], studies have shown that HRR delay can predict cardiovascular risk [5, 7], metabolic risk [5, 6, 29] and worse exercise capacity [5]. Also, HRR may be modifiable with participation in regular physical activity [5].

Some limitations are presented in this study. As this was a study secondary to an RCT, the physical activity level of this sample was not evaluated, which could be analyzed as a possible predictor of HRR. Only two studies evaluated physical activity level and observed better HRV in more active children [7, 29]. Another limitation was the non-evaluation of HR before short agonist bronchodilator (SABA) to determine if there was any influence of medication on resting HR before MST. However, the test was performed at least 30 min after bronchodilator use, which minimizes the effects of the short-acting medication. Additionally, for safety reasons, we could not perform an expected maximal exercise test without SABA, due to the induced bronchospasm exercise risk in asthmatic volunteers.

## Conclusions

In conclusion, children and adolescents with asthma who are under regular treatment have worse HRR after exercise compared to their healthy peers. This information is suggestive of imbalance of autonomic nervous system.

## Data Availability

The datasets during and/or analyzed during the current study available from the corresponding author on reasonable request.

## Abbreviations

%pred: Predicted percentage
μg: Microgram
ACT: Asthma Control Test
AG: Asthma group
ATS: American Thoracic Society
BMI: body mass index
Bpm: Beats per minute
C-ACT: Childhood Asthma Control Test
CG: Control group
CPET: Cardiopulmonary exercise testing
ERS: European Respiratory Society
FEF_25–75_: Forced expiratory flow between 25 and 75% of FVC
FEV_1_: Forced expiratory volume in one second
FVC: Forced vital capacity
GINA: Global Initiative for Asthma
HR: Heart rate
HRR: Heart rate recovery
HRV: Heart rate variability
kg/m^2^: Kilogram per square meter
LF/HF: Low frequency and hight frequency ratio
MST: Modified shuttle test
SABA: Short-acting Beta_2_ agonist
ΔHRR: Heart rate recovery delta
